# A randomized single-blind controlled trial of a prototype digital polytherapeutic for tinnitus

**DOI:** 10.3389/fneur.2022.958730

**Published:** 2022-08-05

**Authors:** Grant D. Searchfield, Philip J. Sanders

**Affiliations:** ^1^Section of Audiology, The University of Auckland, Auckland, New Zealand; ^2^Eisdell Moore Centre, The University of Auckland, Auckland, New Zealand

**Keywords:** clinical trial, tinnitus, digital therapeutic, sound therapy, serious game

## Abstract

**Objective:**

This randomized single-blind controlled trial tested the hypothesis that a prototype digital therapeutic developed to provide goal-based counseling with personalized passive and active game-based sound therapy would provide superior tinnitus outcomes, and similar usability, to a popular passive sound therapy app over a 12 week trial period.

**Methods:**

The digital therapeutic consisted of an app for iPhone or Android smartphone, Bluetooth bone conduction headphones, neck pillow speaker, and a cloud-based clinician dashboard to enable messaging and app personalization. The control app was a popular self-help passive sound therapy app called White Noise Lite (WN). The primary outcome measure was clinically meaningful change in Tinnitus Functional Index (TFI) between baseline and 12 weeks of therapy. Secondary tinnitus measures were the TFI total score and subscales across sessions, rating scales and the Client Oriented Scale of Improvement in Tinnitus (COSIT). Usability of the US and WN interventions were assessed using the System Usability Scale (SUS) and the mHealth App Usability Questionnaire (MAUQ). Ninety-eight participants who were smartphone app users and had chronic moderate-severe tinnitus (>6 months, TFI score > 40) were enrolled and were randomly allocated to one of the intervention groups. Thirty-one participants in the USL group and 30 in the WN group completed 12 weeks of trial.

**Results:**

Mean changes in TFI for the USL group at 6 (16.36, SD 17.96) and 12 weeks (17.83 points, SD 19.87) were clinically meaningful (>13 points reduction), the mean change in WN scores were not clinically meaningful (6 weeks 10.77, SD 18.53; 12 weeks 10.12 points, SD 21.36). A statistically higher proportion of USL participants achieved meaningful TFI change at 6 weeks (55%) and 12 weeks (65%) than the WN group at 6 weeks (33%) and 12 weeks (43%). Mean TFI, rating and COSIT scores favored the US group but were not statistically different from WN. Usability measures were similar for both groups.

**Conclusions:**

The USL group demonstrated a higher proportion of responders than the WN group. The usability of the USL therapeutic was similar to the established WN app. The digital polytherapeutic demonstrated significant benefit for tinnitus reduction supporting further development.

## Introduction

Tinnitus is experienced to some degree by 5–43% of the population depending on definition and the population sampled (1). This false perception of sound can be annoying and can result in, or exacerbate, sleep, concentration, anxiety/depression, and hearing problems (2, 3). Understanding of tinnitus pathophysiology continues to evolve but in general terms, tinnitus can result from disordered or reorganized activity within and across several neural networks due to peripheral auditory deafferentation or head injury (4). Tinnitus magnitude is a complex interaction between detection of the signal, presence of external sound, and influences of attention, memory and emotion (5). Psychosocial factors including personality and environment affect the expression and degree of tinnitus severity (6–8). Tinnitus has unusual perceptual features; it is an unreal or phantom perception which may explain its salience and why distress networks are recruited (9, 10).

The complex nature of tinnitus has so far defeated efforts to develop a medication to eliminate its perception (11). Broad psychology-based management approaches such as Cognitive Behavioral Therapy mitigate some of the negative outcomes of tinnitus (12). There is limited evidence that therapies using hearing aids and sound in a generic manner to mask or facilitate habituation to tinnitus are also helpful (13). Some sound therapies target specific tinnitus generating mechanisms using specialized devices (14) other sound therapies are designed for self-help (15). Despite widespread use, and commercialization, of various forms of sound therapy there has been limited evidence for efficacy, especially in the form of randomized controlled trials (13). Recently several well-designed trials have been published that report the effect of: (1) Tinnitus Retraining Therapy (TRT) compared to partial-TRT and Standard of Care (SOC) (16). (2) Acoustic Coordinated Reset (ACR) T30 Neurostimulator proprietary sound sequence vs. a placebo sound sequence (14). (3) Three bimodal neuromodulation settings combining sound with electrical tongue stimulation (17).

The TRT trial assigned 151 patients to 3 therapies and assessed outcomes at 3, 6, 12, and 18 months (16). At the at end of the study 34 had received and completed TRT, 40 received and completed partial TRT, and 37 received and completed partial SOC. TRT comprised directive counseling and 8 h of sound therapy (18). Partial TRT substituted the normal sound therapy with a “placebo sound therapy” that reduced sound level after 40 min. The SOC was composed of patient-centered counseling and environmental (non-sound generator) sound enrichment. There were few differences between the groups. After 18 months 47.1% of the TRT group, 53.5% of the partial TRT and 40.5% in the SOC group demonstrated a clinically meaningful change in the TFI (>13 points) (16).

Hall et al. (14) compared the Acoustic Coordinated Reset T30 neurostimulator proprietary sound sequence to a placebo algorithm. One hundred and eighteen participants were randomized to the two groups; 44 completed the TFI after 12 weeks of the proprietary sound sequence, 48 completed the TFI after 12 weeks of the placebo algorithm. There were no statistically significant differences in tinnitus measures after 12 weeks of trial. The TFI total score reduced by 1.53 points with the treatment and 3.92 points with the placebo (14).

Conlon et al. (17) tested the effectiveness of 3 different combinations of sound with electrical somatosensory stimulation of the tongue. There was no statistically significant difference between measures for the 3 arms, but all 3 arms showed a clinically meaningful change in average total TFI scores after 12 weeks [arm 1 (*n* = 85) change in TFI 13.9 points, arm 2 (*n* = 88) 13.8 points, arm 3 (*n* = 83) 13.2 points] (17).

The trials described above targeted the neurophysiological processes of habituation (16) neural synchrony (14) and multisensory plasticity (17) in a pre-determined manner across participants. An alternative approach is to apply multiple treatment methods guided by an individual's tinnitus characteristics and therapy goals to focus on aspects of the tinnitus experience likely to be driving other symptoms or preventing adaptation (19, 20). There have been increasing efforts to understand the heterogeneity of tinnitus (21). Through understanding predispositions and environmental factors the possibilities of personalized tinnitus therapy that targets factors critical for tinnitus perception and/or reaction in an individual has been raised (8, 19). The authors' laboratory and clinic have been developing the concept of goal-oriented counseling and Personalized Sound Therapy (19, 22). Our vision is to develop a digital polytherapeutic able to modify multiple different axis of tinnitus perception and reaction, prioritized by individual behavioral needs, tinnitus characteristics and eventually tinnitus biomarkers (22). Methods to measure individual characteristics and goals have been developed (23, 24). Feasibility, proof-of-concept and small randomized trials have investigated potential components of a polytherapeutic including counseling (25) passive sound therapy (26–30) and active training (31–33). From this work a prototype smartphone-based digital therapeutic was developed to provide therapy focused on providing relief, relaxation, and attention focused retraining (34) within the context of counseling focusing on Attention, Reaction, Explanation, and Adaptation [AREA (25)]. This trial will test the efficacy and usability of the prototype tinnitus digital therapeutic and its hardware against a control sound generator smartphone application (app) with earphones commonly used for tinnitus self-help. It was hypothesized that the prototype digital therapeutic would provide superior clinical outcomes with similar usability to the established self-help app.

## Methods

This study was approved by the University of Auckland Human Participants Ethics Committee. All participants gave written informed consent in accordance with the Declaration of Helsinki. This trial was registered on Australian New Zealand Clinical Trials Registry (ANZCTR; ACTRN12621000389808).

### Trial design

The study is a randomized (1:1) parallel two-arm single-blinded controlled study design. The two arms consisted of a prototype tinnitus digital therapeutic developed by the authors and a popular self-help tinnitus app. Repeated outcome measures were obtained at four time points: Screening (week 0), baseline and therapy provision (week 4), 6 weeks with therapy (week 10), and 12 weeks with therapy (week 16). The study ran from 9 March 2021 to 19 March 2022. Participants were seen on week 4 by a single unblinded researcher at a single site, the University of Auckland Clinics, Auckland, New Zealand, all other assessments were undertaken using online materials, in-app notifications, and email reminders. The participants, interventions and procedures undertaken at each appointment and time-frame protocol for data collection are described in the following sections.

### Participants

**Figure 1:**
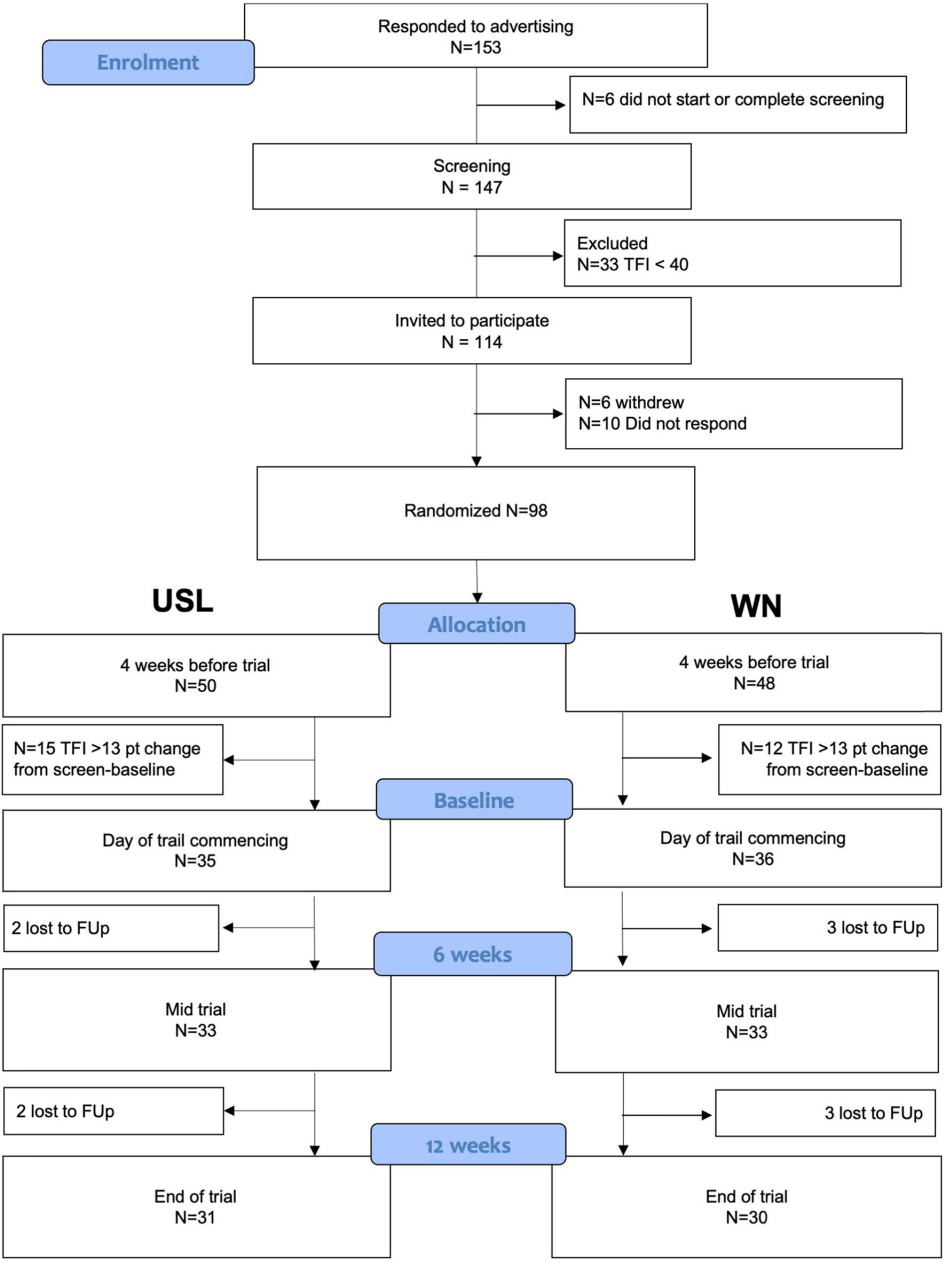
Consort flow chart for participant recruitment and retention.

Participants were recruited by advertisement at a public talk on tinnitus, on the University of Auckland's research website and Facebook. The inclusion criteria were: adults aged over 18, constant tinnitus of at least 6 months duration at baseline, a minimum total score of 40 on the Tinnitus Functional Index [TFI; this cut-off score was chosen as an indicator of moderate-severe tinnitus; (35)], and a maximum of a moderate degree of hearing loss. Hearing aid users were eligible for the study but needed to be able to hear therapy sounds through headphones unaided. Participants had to be smartphone users, be familiar with smartphone apps, and own active Android or Apple phones. Individuals prescribed medications, including for anxiety or depression, were included. Participants were excluded from analysis if their TFI scores changed >13 points (clinically meaningful change) between screening and intervention (indicative of unstable tinnitus or unreliable reporting). Participants were asked to refrain from starting any new tinnitus treatments during the trial. Participants were not reimbursed for participation but were able to keep the apps and headphones provided. The flow of participants through the trial is shown in Figure 1, summary characteristics for enrolled and completer participants are summarized in Table 1 and in detail in Supplementary Table 1.

**Table 1:** Summary of participant characteristics.

	**Per protocol**		**Intent to treat**	
**Measure**	**USL**	**WN**	**USL**	**WN**
Number of participants	31	30	50	48
Age	53 (15)	53 (14)	54 (15)	54 (11)
Gender	19 M 12 F	16 M 14F	28 M 22F	27 M 21F
TFI (Screen)	60 (11)	61 (13)	63(13)	60 (13)
Pitch (Hz)	6,842 (3,698)	5,872 (2,979)	6,419 (3,628)	6,075 (2,808)
Duration	15 (18)	12 (15)	16 (17)	11 (11)
Loudness rating	74 (10)	77 (13)	74 (12)	75 (13)
Awareness rating	71 (23)	76 (21)	73 (25)	71 (24)
Annoyance rating	56 (23)	57 (23)	58 (23)	52 (24)
**Localization**				
Left	5	5	8	6
Right	3	1	3	5
Left of center	4	9	7	10
Right of center	4	3	5	5
Equal ears	10	8	20	16
In head	5	4	7	6
**Hearing loss**				
Yes	22	21	33	35
No	9	9	17	13
**Hearing aids**				
No	24	26	38	42
Left	1	1	1	3
Right	2	0	2	0
Binaural	4	3	9	3

### Interventions

#### Active control

**Figure 2:**
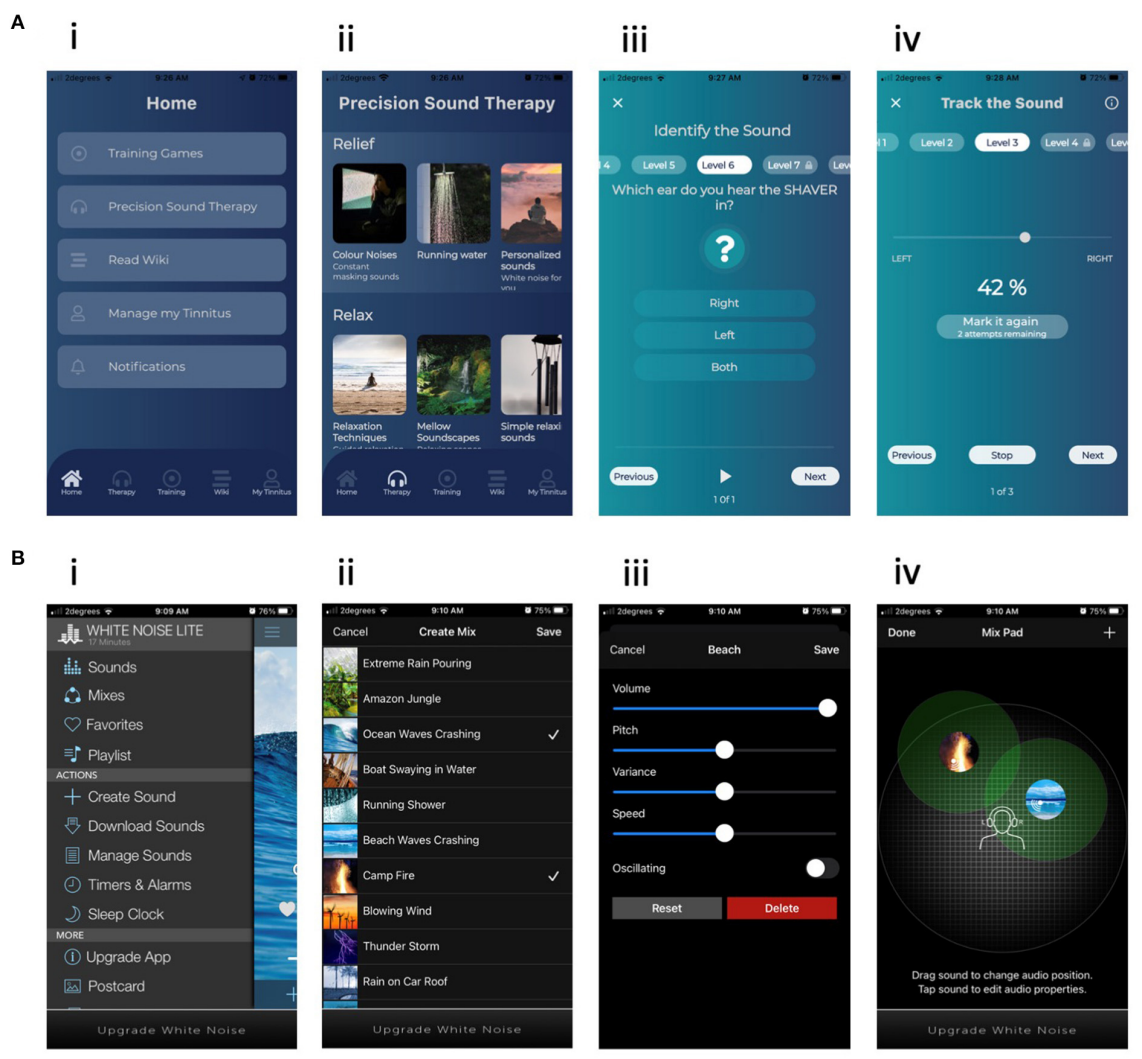
Example screenshots for **(A)** the USL intervention (i) Menu, (ii) Passive therapy sounds, (iii) AOIL task, (iv) Tracking task. **(B)** The WN intervention. (i) Menu, (ii) Passive therapy sounds, (iii) Sound control, (iv) Sound mixing.

The active control was the “White Noise” (WN, TMSOFT) app, available from the Play Store (Google) and App Store (Apple). Example screen shots of the user interface are shown in Figure 2B. Participants were provided in-ear wired headphones (e.g., Panasonic RP-HJE290GUK Premium Black Earphones) but were also free to use their own headphones of any type if preferred. WN was chosen as the active control as it was available across platforms and resembled the test intervention in use of sound and phone, and has previously been identified as a popular self-help app for tinnitus (15). All participants had a range of sounds available to access based on personal preference. The clinician did not customize the control app. Participants were shown functions on the app such as timers and sound manipulation capabilities (location, volume etc).

#### Digital therapeutic

For the purposes of the trial this was given the name “UpSilent” (USL, F-Code labs) (Figure 2A). The therapeutic consisted of a smartphone app, Bluetooth bone conduction headphones (Z8, Shenzhen JEDI Technology Co) and Bluetooth neck pillow speaker (U-shape, Shenzhen Epoch Technology) for sleep, and written counseling materials. The researcher had partial control over the overall system and could remotely enable or disable functionalities, modes and content of the patient's app using a cloud-based clinician dashboard. A customized profile was chosen according to the patients' needs and tinnitus assessment. The app had three different modes 1. Passive listening (Relief, Relax, & Retraining sounds). 2. Active listening and 3. Counseling (AREA).

##### Passive listening

The tracks were selected from a library of sounds according to participant preference and goals. Relief sounds had high interaction with tinnitus creating masking, a personalized sound where the frequency response was tailored to the individuals minimum masking levels (36) and perceived position in space (29) was included. Relaxation sounds had positive emotion affect associated with calm situations (e.g., gentle waterfall). Retraining sounds were more complex nature sounds with multiple sound objects and participants were instructed to focus attention on these sounds, enabling retraining of attention away from tinnitus.

##### Active listening

This consisted of two components a tinnitus calibration task (23) and a form of the Auditory Object Identification and Localization (AOIL) task (31). The calibration task gave the player agency over a sound like their tinnitus and encouraged moving attention in auditory space away from their tinnitus. Participants had to listen for the location of a target tinnitus avatar sound and use a slider to manipulate the location to match the location of the target. The AOIL was an attentional training program. Participants were presented with a variety of different “everyday” sounds monaurally or binaurally. Participants were instructed to attend to given locations or sounds, and respond to prompts (e.g., “Which ear did you hear the SHAVER in?”). Feedback was provided on correct/incorrect identification.

##### Counseling

Brief psychoeducation following the AREA model (25) was provided consisting of a Wiki about tinnitus and how to use the UpSilent sounds to enable therapy strategies. Strategies included goal setting, sleep hygiene, attention control techniques, communication strategies, guided abbreviated progressive relaxation and deep breathing exercises.

### Procedures

Participants were blinded as to the intervention they received. The researcher providing the therapy could not be blinded. The number, duration and content of research sessions were the same for both arms to control for non-specific effects of the device, care, and therapeutic attention. The participants were instructed to use the interventions as needed and to aim for a minimum of 2 h of cumulative use per day for 12 weeks. The instructions per participant varied as part of the goal setting and needs assessment process. Participants requiring “relief” were recommended “relief” sounds until some control over tinnitus was achieved, those for whom relaxation was an important goal were recommended those sounds when stressed. Following relief and relaxation, participants were recommended to focus on retraining strategies.

All participants were provided verbal counseling on the use of sound therapies for tinnitus according to the goals and needs established through information provided in their enrolment questionnaires and discussion with the researcher at the start of the appointment. All were provided with generic information around tinnitus and its pathology.

#### Screening (week 0, online)

Following contacting the researchers, participants were provided with an information sheet that outlined the background and aims of the trial and details of measurements to be taken over the course of the study. After providing written informed consent, participants were assigned a unique identifier code so that data was managed and analyzed in a deidentified manner. Participants were provided with a link to online questionnaires coded, stored, and collated using the University's REDCap (Research Electronic Data Capture) account. The system operates in accordance with safe design and software maintenance standards for medical software. Participants completed a comprehensive case history [Tinnitus Sample Case History Questionnaire, TSCHQ (37)]. The TFI, a recognized tinnitus intake and assessment questionnaire (35) validated in New Zealand (38) was completed. The TFI served as the primary outcome measure in this trial. The TFI consists of 25 items and eight subscales, where a 0–10-point Likert scale measures the response to each item. The subscales address the domains where the tinnitus impacts the patient (35). Participants were asked how much a problem their tinnitus was (0 not a problem−5 very big problem). Numeric rating scales were used to measure tinnitus perception along five dimensions: How strong, intrusive, uncomfortable, unpleasant the tinnitus signal was, and how easy it was to ignore the tinnitus signal (0–10 rating, 0 not a problem−10 extreme problem).

#### Randomization

Participant allocation (1:1) to each study arm was randomized using a computer random number generator.

#### Baseline (week 4, in person)

Following a 20-min period of active listening about the individual's tinnitus, assessments were undertaken by the researcher. Participants needs and goals with therapy were ascertained using the Client Oriented Scale of Improvement in Tinnitus (COSIT) (24). The COSIT is an open-ended questionnaire in which the participant listed up to five improvement goals they hoped to realize with the therapy, that they then ranked. In addition to active listening and goal setting, in-person counseling for both groups were limited to description of the therapy goals and instructions on device use.

Outcomes were assessed at the end of the trial (week 16) as to degree the therapy had changed their tinnitus, and its final status relative to goals. The TFI and rating scales were undertaken online.

Pure tone audiometry (MEdRX, AVANT Stealth Audiometer, 0.25–16 kHz) was conducted in a sound treated room (ISO 8253–1:2010) and employed the modified Hughson-Westlake procedure (39). Tinnitus psychoacoustic outcomes were measured using tinnitus testing software (MEdRX, Tinnometer). Tinnitus pitch match was assessed throughout the test frequency range of 0.25–16 kHz using a two-alternative forced-choice method. Pitch match was then compared to tones one octave above and below to rule out octave confusion. The measurement was repeated until two repeatable responses were obtained.

#### Fitting process

Participants in both arms worked with the researcher to create a personalized sound using the Threshold Adjusted Noise (TAN) method (36) with Adobe Audition software. In this method white noise is filtered through a graphic equalizer with frequency band levels adjusted according to hearing thresholds and minimum masking levels at frequencies between 0.5 and 8 kHz using a modified Hughson-Westlake procedure (36). The participant's preferred sound location was then ascertained (23). The Anaglyph plugin (40) within Adobe Audition software was used to simulate the TAN sound moving around the head, using the numbers of the clock relative to the head as points of reference (e.g., 12 o'clock is directly in front, 3 o'clock is over the right ear) to create a spatialized version of the personalized TAN sound. The personalized sounds were later available through the app to the US arm only.

The participants were familiarized to the intervention they were assigned. The researcher helped to download and install the relevant app, and instructed everyone on its use, as well as the associated hardware (BC headphones and neck speaker for USL). Each group received instruction from the researcher on the functions available in the relevant intervention (USL or WN) and were provided with a written manual for the appropriate app outlining these functions.

#### Mid trial 6 weeks of therapy (week 10, online)

The TFI and rating scales were repeated.

#### Completion 12 weeks of therapy (week 16, online)

The TFI and rating scales were repeated. COSIT outcomes were ascertained. Usability of the US and WN interventions were assessed using the System Usability Scale [SUS, (41)] and mHealth App Usability Questionnaire for Standalone mHealth Apps used by Patients [MAUQ-SPA, (42)]. The SUS is a 10-item scale widely used in usability engineering with 5 response options: 1 (strongly disagree) to 5 (strongly agree). The MAUQ-SPA is an 18-item scale requiring responses from 1 (strongly agree) to 7 (strongly disagree).

### Compliance

Compliance with use of the interventions were self-reported in free-field sections of an end-of-treatment questionnaire. Additional monitoring was completed by the researcher at assessment times through email. Participants were monitored for adverse effects.

### Interim analysis and stopping rules

There were no interim analyses or stopping rules for the trial.

### Statistics

A power analysis calculation was undertaken (G^*^Power 3.1) to determine the sample size for a repeated measures between factors ANOVA with two groups and 4 repeated measures. For an effect size of 0.3 an alpha of 0.05 and power 0.95 a sample size of 94 was calculated. Recruiting 100 participants (50 per group) allowed for a dropout rate of 5%. Intent-to-treat and completer (per protocol) analyses where undertaken. Completer analysis limited data analysis to those participants that undertook all evaluations as per the protocol (*N* = 61), so measures of change represent changes within individuals, data examined include the TFI total and subscales, rating scales, COSIT scores, and SUS and MAUQ scores. Per-protocol (completer) analysis was chosen as primary method as COSIT, SUS, and MAUQ are only completed at trial end. The demographics of all enrolled participants are present alongside intent-to-treat analysis for the TFI total score to confirm the primary per-protocol analysis was unbiased.

#### Baseline measures

**Figure 3:**
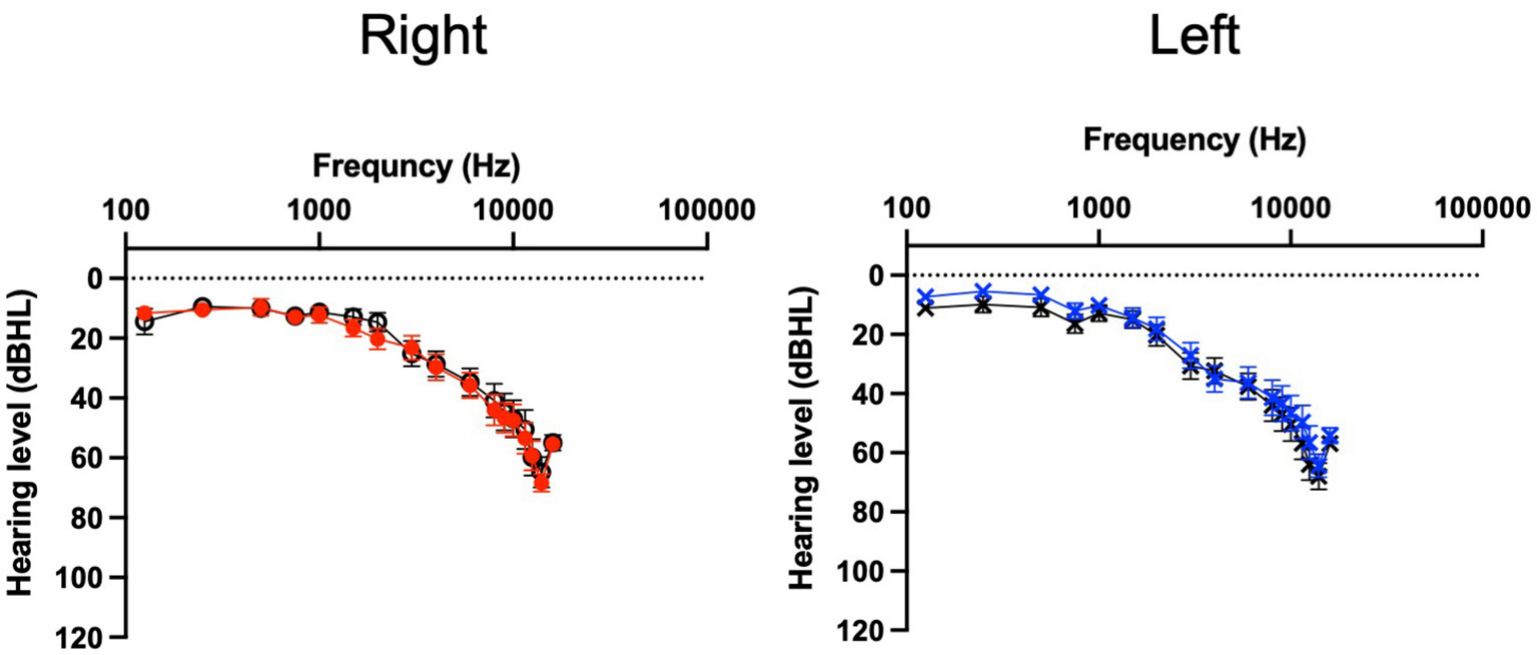
Audiogram for per protocol participants. USL group (color, *n* = 31) WN group (black, *n* = 30). Mean thresholds and standard error bars are shown.

Analysis of data was undertaken using GraphPad Prism 9.3.1 for Mac. Means, standard deviations (SD) and proportions were used to describe the baseline characteristics of study participants (Table 1). Baseline data was not normally distributed and often categorical. The Mann-Whitney test was used with the Holm-Šídák method for multiple comparisons between USL and WN for the baseline measures and for audiometry (Figure 3).

#### Outcomes

The primary outcome measure was a responder analysis of the proportion of participants with clinically meaningful change in TFI (>13-point change, baseline to 6 and 12 weeks of intervention) between the 2 groups. Secondary analyses were within and between group differences in: TFI total score, TFI subscales and rating scales baseline across time. COSIT, SUS and MAUQ scores were compared between groups. The normality assumption was confirmed using the Shapiro-Wilk test for TFI, COSIT, SUS, and MAUQ data. Data for rating scales and the COSIT were not normally distributed.

Chi-square contingency testing was undertaken to test that the proportion of participants with clinically meaningful change in the TFI was greater for the USL group than WN group as a responder analysis. Proportional differences grouped according to degrees of change were explored for baseline to 12-week data. The hypothesis that the TFI total score would be different between groups from baseline to 12 weeks was tested using an unpaired *t*-test. A two-way ANOVA with Geisser-Greenhouse correction was used to analyse TFI Total and subscale data for per-protocol analysis. Dunnett's multiple comparisons test was used to compare screening, 6 and 12 week scores to the baseline score within USL and WN arms. A mixed effects ANOVA (Split-plot ANOVA) with Geisser-Greenhouse correction was used to analyse TFI data in the intention to treat analysis due to missing data. Within arm effect sizes (Cohen's *d*) were calculated for the intention to treat scores to enable comparison with previous studies as the mean score at 6 or 12 weeks of treatment minus the mean score at baseline divided by the pooled SD. The Friedman test was used for non-normally distributed measures (ratings) with Dunn's method for multiple comparisons of screening, 6 and 12 week scores to the baseline score within USL and WN arms. SUS data for USL and WN were analyzed using a one-way ANOVA. COSIT and MAUQ data for the groups were compared using unpaired *t*-tests.

## Results

### Participant characteristics

The flow of participants from contacting the researchers through to study completion are shown in Figure 1. The characteristics of participants completing the study (per-protocol) and at enrolment (intent-to-treat) are summarized in Table 1 and pure tone audiometry is shown in Figure 3. Additional characteristics of the population are provided in Supplementary Table 1 and Supplementary Figure 1. Both the USL and WN groups within and between per-protocol and intent-to-treat groups were similar. Thirty-one individuals in the USL group [age 53 years (SD 15), 19 male 12 female, screening TFI 60 (SD 11)] and Thirty in the WN group [age 53 (SD 14), 16 male 14 female, screening TFI 61 (SD 13)] completed all aspects of the study and were the primary focus of analysis.

### Responder analysis

**Figure 4:**
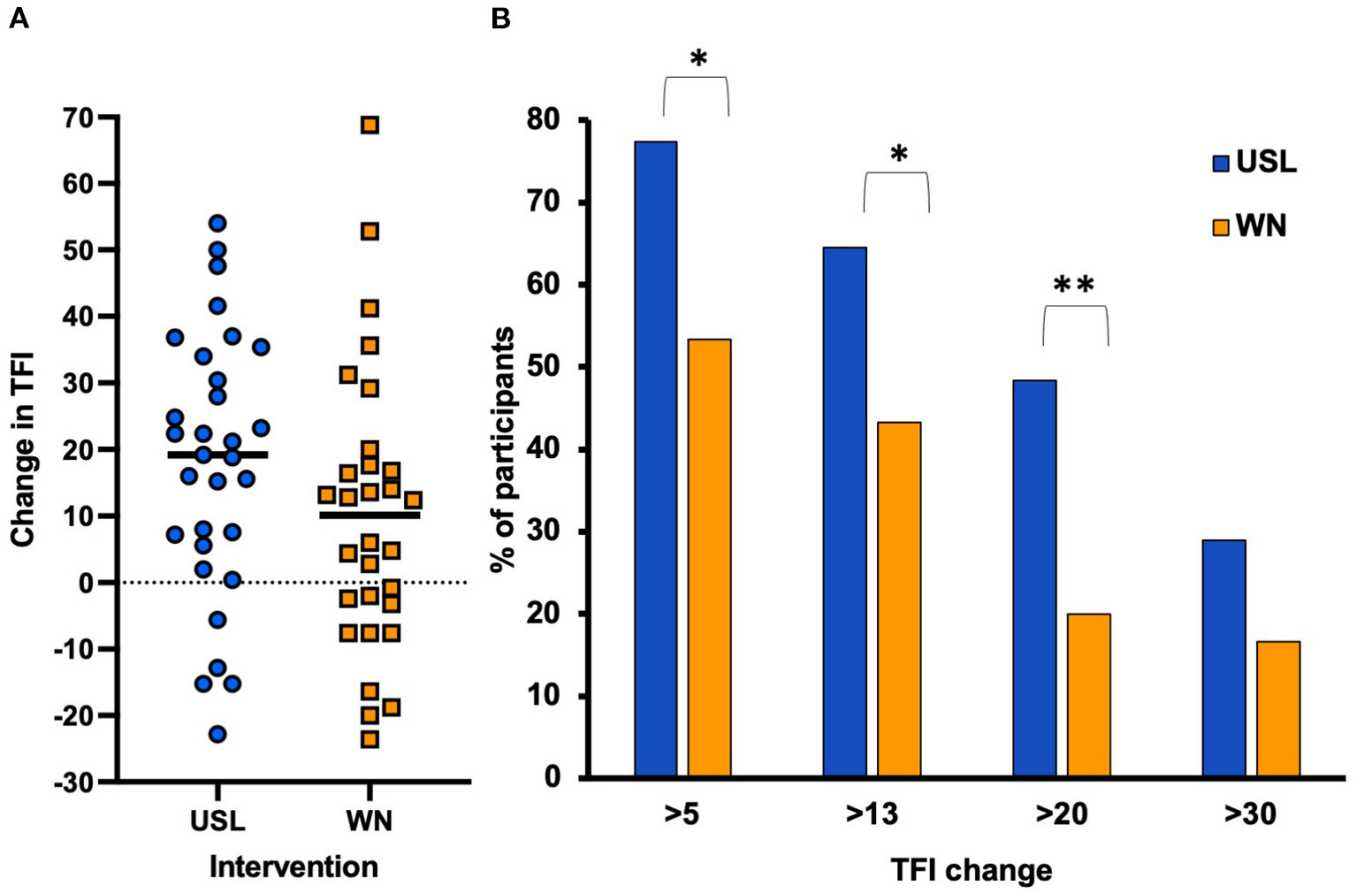
**(A)** Change in TFI total score between baseline and 12 weeks for each group. The horizontal line indicates average TFI change for each group. **(B)** Responder analysis. The proportion of the two groups with reduced TFI of (>5, 13, 20, and 30 points) at 12 weeks of trial (*P < 0.05, **P < 0.01). A change of >13 points is considered clinically meaningful.

The average change in total TFI score between baseline and 12 weeks was 17.83 points (SD 19.87) for the USL group and 10.12 points (SD 21.36) for the WN group (Figure 4A). A clinically meaningful change in total TFI score is considered 13 points or more. A statistically greater proportion of USL participants (55%) had a meaningful change in total TFI with 6 weeks of intervention (16.36, SD 17.96) compared to WN (33%, 10.12 points, SD 18.53) (χ^2^ = 2.858, *P* = 0.046). At 12 weeks a statistically greater proportion of USL participants had a meaningful change in total TFI (65%) compared to WN (43%) (χ^2^ = 2.775, *P* = 0.049). The proportions of responders using criteria of 5 to 30 points change were calculated. There were a higher proportion of responders for greater change than 5 points (χ^2^ = 3.918, *P* = 0.024) and 20 points (χ^2^ = 5.442, P=0.01) but not 30 points (χ^2^ = 1.318, n.s) (Figure 4B).

### TFI mean values

**Figure 5:**
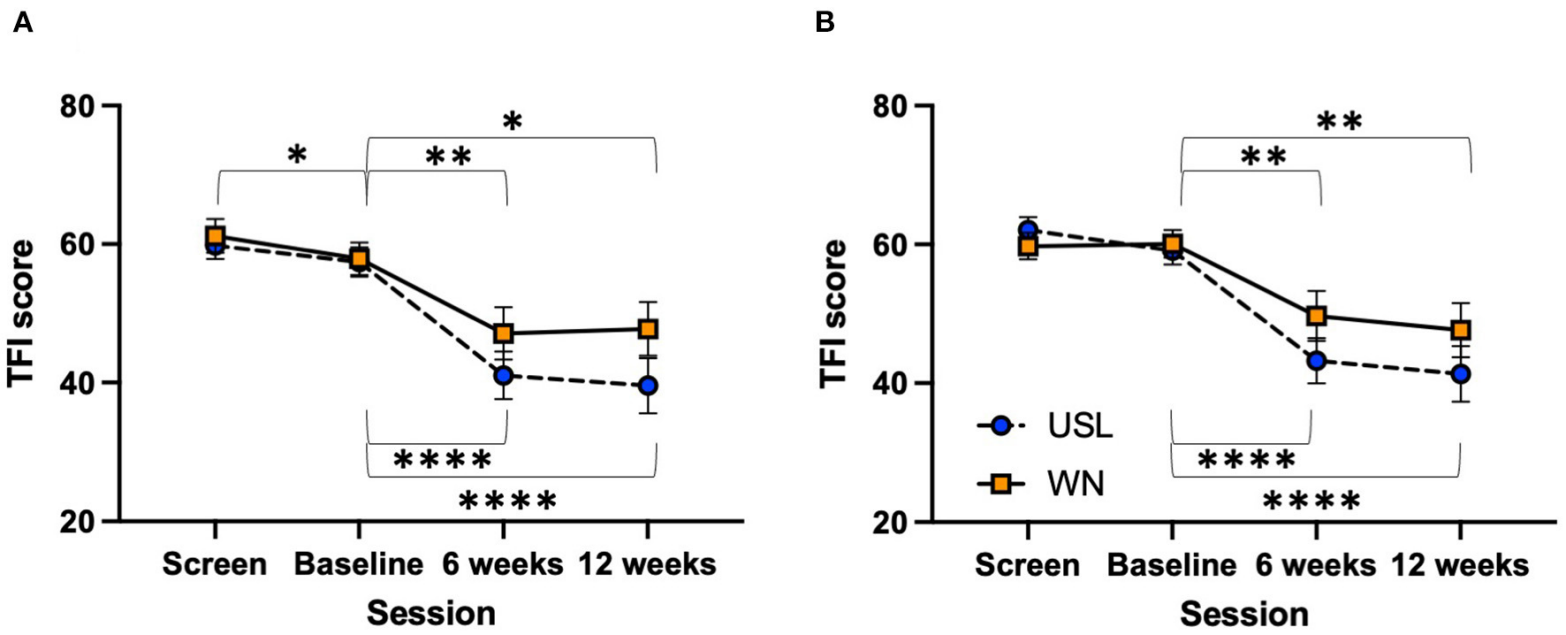
TFI score across sessions. **(A)** Per-protocol. **(B)** Intent-to-treat. USL group (dashed line) WN group (solid line) (*P < 0.05, **P < 0.01, ***P < 0.001, ****P < 0.001). Mean scores and standard error bars are shown.

**Figure 6:**
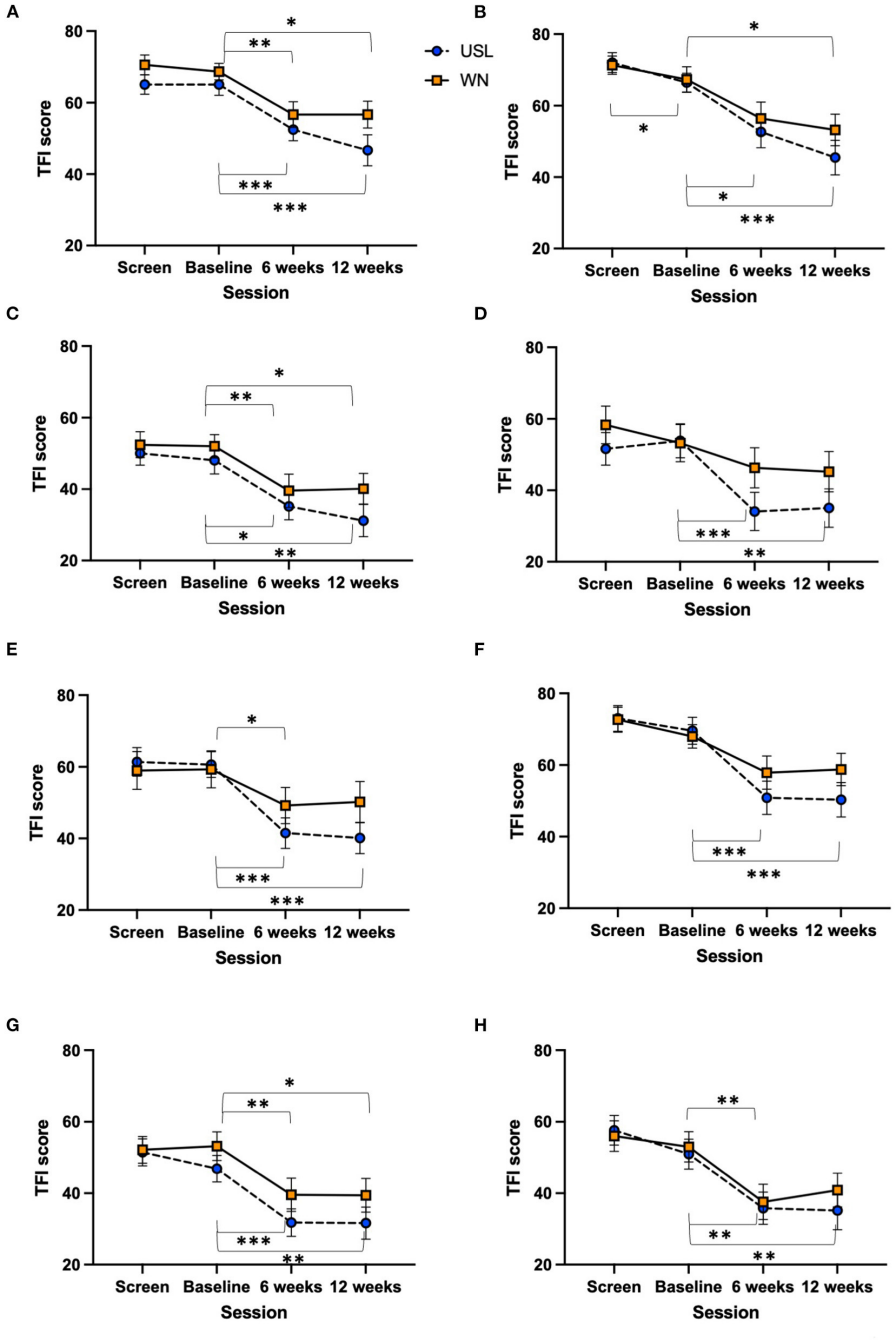
TFI subscale scores across sessions. **(A)** Intrusive, **(B)** control, **(C)** cognitive, **(D)** sleep, **(E)** auditory, **(F)** relaxation, **(G)** quality of life, and **(H)** emotional distress. USL group (dashed line) WN group (solid line) (*P < 0.05, **P < 0.01, ***P < 0.001). Mean scores and standard error bars are shown.

The change in total TFI score between baseline and 12 weeks of intervention for each completing participant group (USL *N* = 31, WN *N* = 30) was analyzed. The TFI, and subscales, were normally distributed. There was no statistically significant difference in the change of TFI score from baseline to 12 weeks of intervention between the USL and WN groups [*t*_(59)_ = 1.461, n.s]. TFI scores within groups across time for the TFI total and subscale scores were explored. Using a Two-way ANOVA there was a significant main effect of session [*F*_(1.603,94.57)_ = 34.88, *P* < 0.0001] across four measurement times but no session by group interaction [*F*_(3,177)_ = 1.516, n.s] (Supplementary Table 2). Within group comparisons using Dunnett's multiple comparison test identified statistically significant differences between sessions for both groups (Figure 5A, Supplementary Table 3). Equivalent results were found for the intent-to-treat analysis using a mixed measures ANOVA [*F*_(1.799,115.7)_ = 23.66, *P* < 0.0001] with no session by group interaction [*F*_(3,193)_ = 1.595, n.s] (Figure 5B). Each subscale of the TFI was assessed using ANOVAs (Figure 6, Supplementary Table 2) and *post-hoc* Dunnett's multiple comparison test in which screening, 6 and 12 week sessions were compared to baseline (Supplementary Table 3). There were significant main effects for session for all subscales. In the case of the Auditory Subscale *F*_(2.258,133.2)_ = 22.24, *P* < 0.0001 there was a significant session by treatment interaction *F*_(3,177)_ = 3.020, *P* = 0.0312 (Figure 6E). This interaction was explored further with multiple *t*-tests using the Holm-Šídák correction for multiple comparisons, the values for USL and WN were not statistically different. Within group comparisons using Dunnett's multiple comparison test identified statistically significant differences between sessions for both groups for most subscales (Figure 6, Supplementary Table 3). Significant differences within group for the Sleep (Figure 6D) and Relax (Figure 6F) subscales were found for the USL group but not the WN group.

### Rating scales

**Figure 7:**
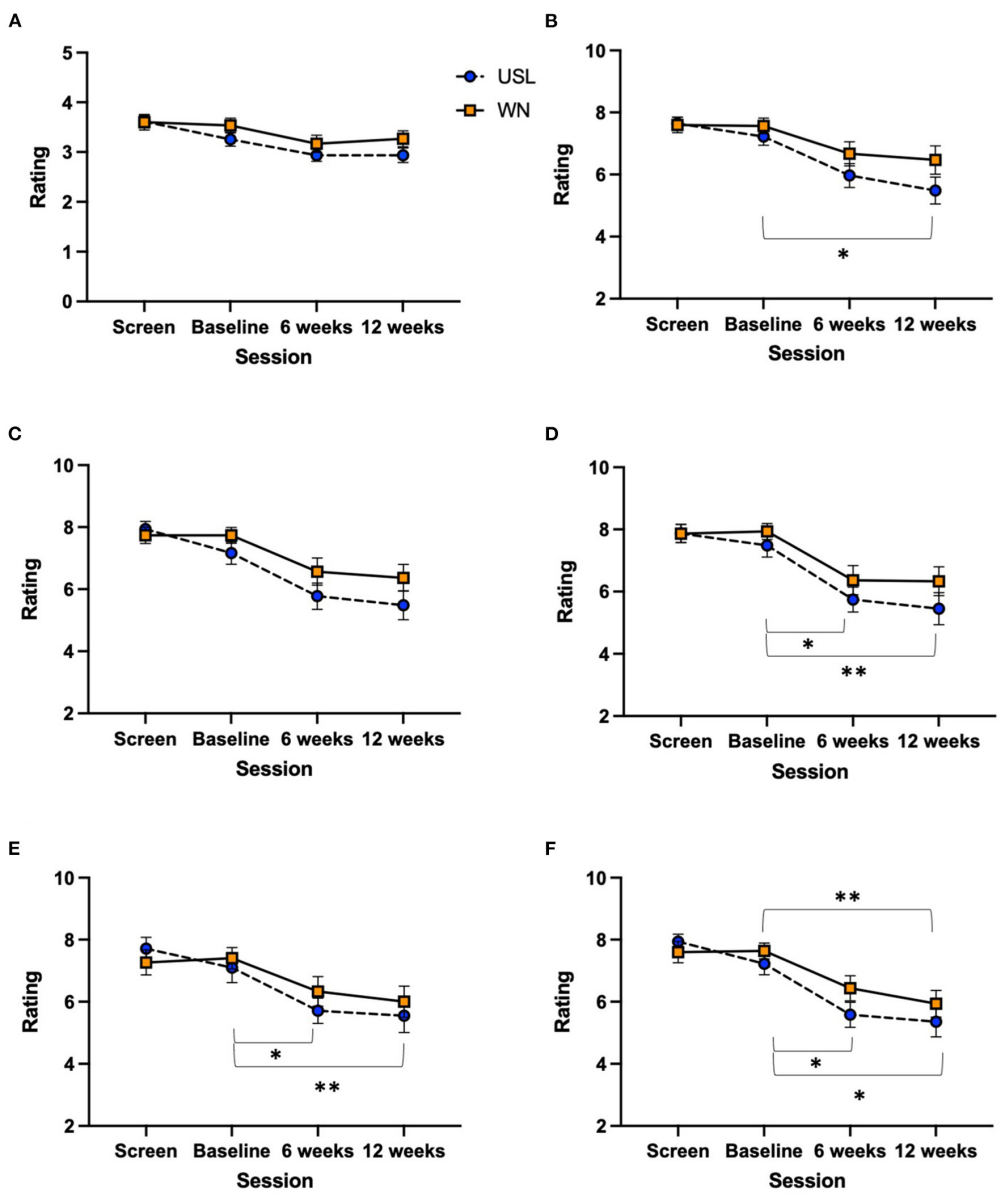
Rating scales scores across sessions. **(A)** Problem, **(B)** strong, **(C)** uncomfortable, **(D)** annoyance, **(E)** ignore, and **(F)** unpleasant. USL group (dashed line) WN group (solid line) (*P < 0.05, **P < 0.01). For consistency with other figures mean and standard error scores are shown, the non-parametric statistics shown are based on rankings.

Rating scales were not normally distributed and so the non-parametric Friedman test (Supplementary Table 4) was used to explore within intervention effects. Dunn's multiple comparison test was used to compare screening, 6 and 12 week sessions to baseline (Supplementary Table 5). There was a main effect of session for USL and WN groups. *Post-hoc* Dunn's tests identified significant differences in the USL group between baseline and post intervention sessions for Strong, Annoyance, Ignore and Unpleasant rating scales (Figure 7). Dunn's tests identified significant differences in the WN group between baseline and 12 weeks of intervention for the Unpleasant rating scale (Figure 7F).

### COSIT and usability

**Figure 8:**
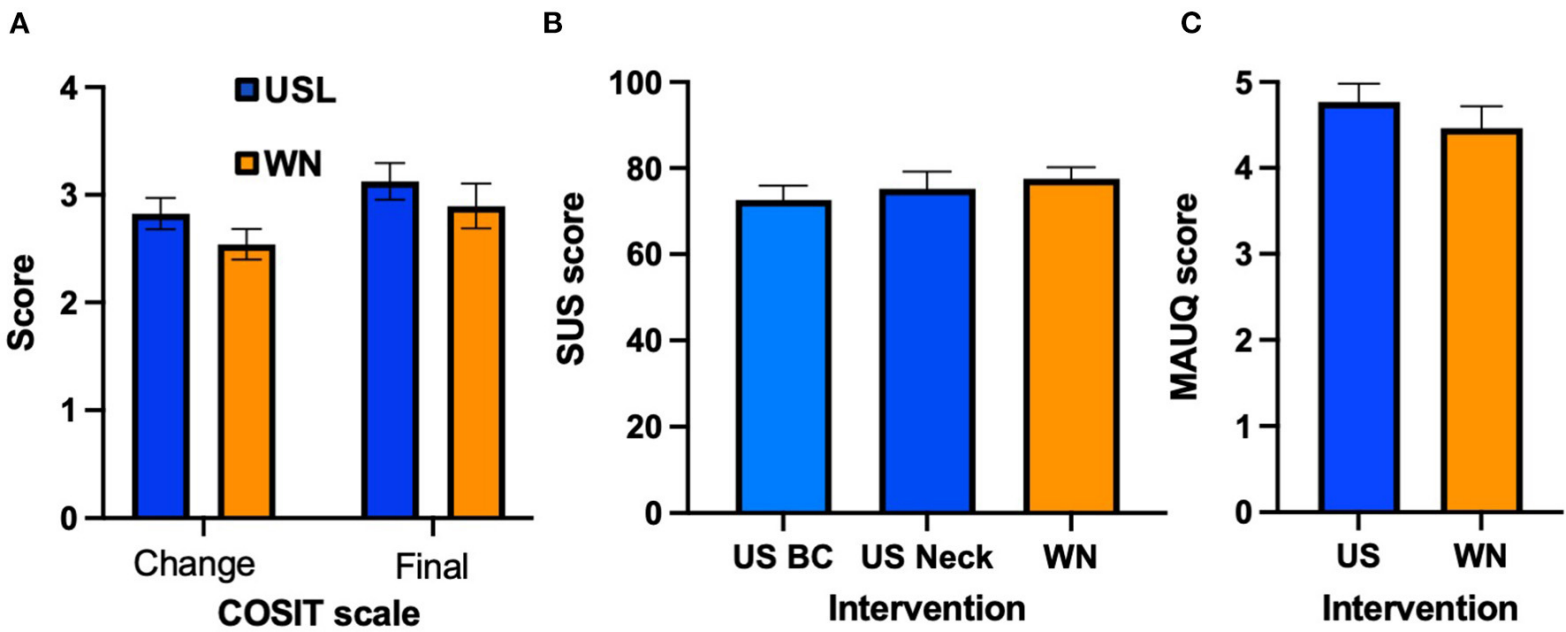
**(A)** COSIT scores (USL group, Blue; WN group, Orange). **(B)** System Usability Scale (SUS) scores. **(C)** MHealth app usability questionnaire (MAUQ) scores. Mean scores and standard error bars are shown.

The COSIT scales were analyzed using a 2-way ANOVA to compare data from USL and WN groups for the 2 COSIT scores. The groups did not differ significantly [*F*_(1,118)_ = 0.02997, n.s, Figure 8A]. The COSIT degree of change score “With the therapy my tinnitus is…” ranges from 1 worse−2 no different−3 slightly better−4 better−5 much better. The USL group change score was 2.83 (SD 0.82) the WN group was 2.54 (SD 0.78). The COSIT final score “I am annoyed by the tinnitus…” ranges from 1 almost always−2 most of the time−3 half of the time−4 occasionally−5 hardly ever. The USL group final score was 3.13 (SD 0.95) and for the WN group it was 2.90 (SD 1.14).

Data for the SUS and MAUQ were normally distributed. A one-way ANOVA found no statistically significant difference [*F*_(2,89)_ = 0.519, n.s] between SUS scores for USL with BC headphone (72.66, SD 18.20) USL with pillow speaker (75.24, SD 21.86) and WN (77.50, SD 14.87) (Figure 8B). No statistically significant difference [*t*_(56)_ = 0.922, n.s] was found between MAUQ scores for USL (4.77, SD 1.16) and WN (4.47, SD 14.87) (Figure 8C).

### Effect size

**Figure 9:**
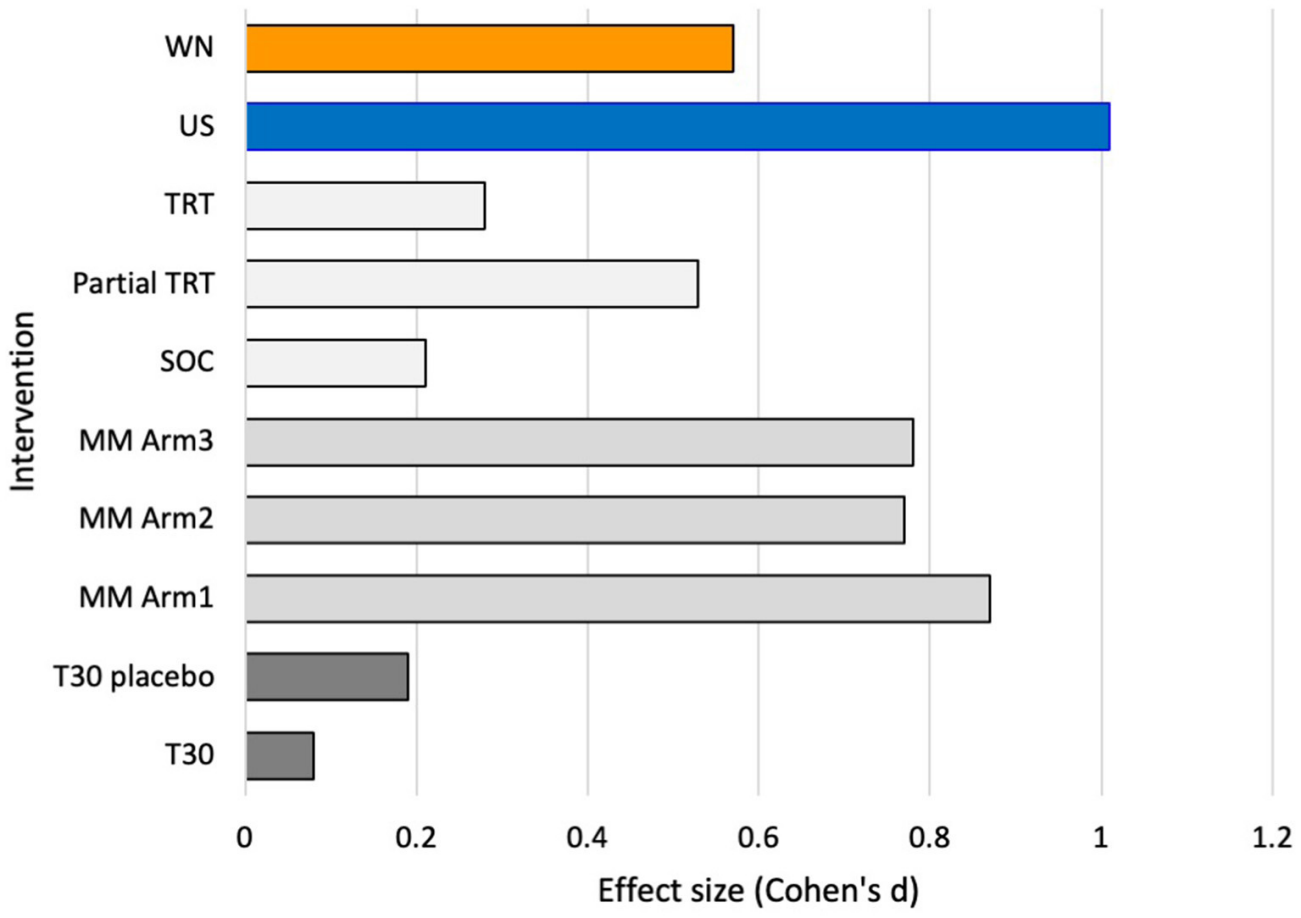
Effect size (Cohen's *d*) for recent clinical trials at 12 weeks (3 months) of intervention. T30 stimulator trial (14), Multimodal (MM) sound and tongue stimulation trial (17), Tinnitus Retraining therapy (TRT) Partial TRT and Standard of Care trial (16), and current trial (WN, USL, intent-to-treat data).

The Cohen's *d* effect size at 12 weeks for intent to treat analysis was 1.01 for USL and 0.57 for WN; the Cohen's d effect size for TFI results across a common time reported by Conlon et al. (17), Hall et al. (14), and calculated from Scherer and Formby (16), are shown in Figure 9.

### Compliance

For those participants who completed the study per protocol: 1 participant (USL) reported that they did not use the intervention, 2 (USL) reported inconsistent use, 4 (USL = 1, WN = 3) reported minimal use (e.g., “not used much,” “1–2 day a week, briefly”), 2 (USL = 1, WN = 1) stopped using the intervention within the first 2 weeks of the appointment, 4 (USL = 3, WN = 1) stopped after 6 weeks, 1 (WN) stopped after 9–10 weeks, 14 (USL = 10, WN = 4) initially used the intervention for 2 h per day, but use declined over the duration of their participation in the study, 11 (WN) used the intervention daily for <2 h, 22 (13 = USL, 9 = WN) used the intervention for at least 2 h per day.

## Discussion

Both treatment groups demonstrated reductions in tinnitus from baseline measures after 12 weeks of therapy. The USL intervention provided superior outcomes across most measures. Changes in TFI for the USL group at 6 and 12 weeks were clinically meaningful whereas the mean changes for WN were not. A responder analysis showed a statistically higher proportion of USL participants achieved meaningful TFI change than the WN group at 6 and 12 weeks. Statistically significant differences to baseline were found within therapy for “strong,” “sleep,” “auditory,” and “relaxation” ratings for the USL group and “relaxation” rating for the WN group. Numerical changes in mean TFI and subscales within groups were larger for the USL than for the WN group, but there was not a statistically significant interaction, both groups showed improvement. There were no statistically significant differences in the COSIT or usability measures. The responsiveness of the COSIT to intervention has not been psychometrically evaluated (24). Although the usability of the interventions were assessed as equivalent, participants using the prototype did report software bugs, especially when their phone operating system was updated.

These results indicate the strong potential of a treatment based on the prototype used here. Responder analysis using the standard >13-point TFI change as criteria for clinically meaningful change demonstrated greater success of the USL intervention. Exploring different criterial for change (38) was consistent with USL superiority. Superiority is not often found in studies comparing treatment settings or different treatments (14, 16, 17). Effect size is another indication of the size of any effect and in the case of USL this was found to be large. A comparison with several recent well-constructed trials using the TFI suggest the relative potential of the USL prototype (Figure 9). The effect size for USL was the largest compared to the equivalent studies sampled. The WN passive sound therapy was similar to partial TRT which it closely resembles consisting of counseling and passive sound therapy for a limited time period (16).

This study demonstrates the benefits of the USL prototype digital polytherapeutic. It was not designed to identify which of its therapeutic components or hardware was most responsible for effect. The USL and WN interventions both resulted in within treatment statistically significant changes to the various TFI subscales. Fewer changes were observed in the rating scales, so these perhaps provide a useful, tentative, indication of modes of effect. Rating scales may be less responsive to change; consisting of a single measure, as opposed to several (such as the TFI subscales) and being a snapshot in time (in contrast the TFI asks what the effect is “over the past week”). It is possible that for the rater to indicate a change on a simple scale the treatment effect must be larger. The only statistically significant change from baseline for WN was the rating of unpleasantness being reduced between baseline and 12 weeks of therapy. USL had significant effects on ratings of annoyance, ability to ignore and unpleasantness between baseline and 6 weeks of therapy; between baseline and 12 weeks of therapy ratings of annoyance, ability to ignore and unpleasantness remained significant, and the strength of tinnitus (loudness) had also reached threshold for statistical significance. Scales of problem and comfort did not change statistically. The results are consistent with a rapid positive effect that is broadly based. An ecological model of tinnitus that incorporated Adaptation Level Theory proposed that a multitude of inherent, and environmental factors interact to determine final tinnitus magnitude (8). Tinnitus and external sound interact and undergo similar auditory processing within the system, including feature extraction, schema formation, and semantic objective formation (8, 31). Informational or “central” masking is possible with tinnitus, as the phenomenon is due to central processing itself (34). Another way in which sounds can promote relief is by positive affect (43). The final magnitude estimates of tinnitus, as well as distress judgements, are derived by interactions between the tinnitus, contextual components (any background sound), and cognitive-behavioral characteristics such as personality traits, memory, and past experiences, and emotion (6). According to the Adaptation Level Theory of tinnitus (5) therapeutic benefit can be achieved by increasing the focus on, and driving, non-tinnitus neural activity. This can be achieved through a combination of attention re-focusing counseling alongside active and passive sound therapy. According to this theory, reductions seen in tinnitus perception occur through the experiential learning of a new adaptation level. Tinnitus might not be perceived if tinnitus falls below the individuals signal detection threshold (5).

The study had several strengths, and some limitations. The study assessed tinnitus across time and used multiple measures. The assessment dimensions used are consistent with the recommendations for core outcome measures in assessing sound-based therapies (44). Two of the outcome measures the TFI (38) and COSIT (24) have also been evaluated in the NZ tinnitus population, providing confidence that the outcomes measured are valid. Participants were blinded to the intervention. All participants received the same assessment, including processes for customization of sound (only employed in the USL app). Instructions and contact with the research were similar. The comparison intervention was an active control, it was anticipated to provide benefit, we hypothesized that the USL intervention would be superior. The WN app had a similar look to the USL intervention, and it employed the users' Smartphone in a similar way, so controlled for influence of being provided with technology. The researcher was not blinded, so there was a risk of unintended bias. The decision not to blind the research was a pragmatic decision based on the need that they dispense the therapy. The researchers contact with participants in both groups was limited to the one assessment and dispensing session, with screening and follow up assessments being undertaken online. The researcher undertook technical troubleshooting and was available to answer participant questions from participants in both groups.

Although mean outcome values were numerically different, variance in response indicates that larger sample sizes are needed. This research took place during the severe acute respiratory syndrome coronavirus 2 (SARS-CoV-2) pandemic when New Zealand was subject to various lockdowns that restricted recruitment. A power analysis was undertaken apriori and indicated 47 participants in each group. We anticipated a 5% reduction in participants from enrollment to study completion. The actual amount was 38%; other reference studies analyzed 21% (16), 15% (14), and 50% (17) fewer participants at 12 weeks than first enrolled. Our numbers were largely driven by a high proportion of participants with clinically meaningful fluctuation between screening and baseline (28%). The standard statistics indicate whether differences observed are due to chance. This will be accounted for in future study design by our group. This finding serves to highlight the value of a no intervention baseline period to identify fluctuating tinnitus or unreliable observers. We recommend that other trials use this approach as it may reduce non-intervention variance. The research was undertaken at a single site by the developers of the USL therapy, this carries the risk of unconscious bias. Future trials should include multiple sites independent of the developers.

The presence of several influencing factors on tinnitus-external sound interactions might account for individual success (or lack of success) with the US therapy compared to the WN app. The difference in the interventions included use of different hardware. The selection of hardware was based on testing the digital therapeutic system as a whole against the normal use of headphones with an app. It is possible that the hardware accounts for some of the differences seen between groups. Future testing of different parameters, hardware and individual preferences for sound therapy will be important to strengthening evidence for, and improving, the treatment effectiveness (45).

A goal of future iterations of the therapeutic is to further empower the individual with a sense of greater control over their tinnitus. We believe that greater personalization and interaction in therapy selection (including therapeutic sounds) will enhance this sense of control. At present the goal-focused approach using the COSIT provides individualization through prioritization of therapy module use. AI to aid this through prediction of effective treatment and preference-based learning is being developed (22).

## Conclusions

Both therapies trialed provided benefit. The US therapeutic resulted in clinically meaningful change in a larger proportion of participants and a large treatment effect. The intervention tested in this research is a step toward an effective digital polytherapeutic that can accommodate individual goals and predictors of therapy success by employing multiple strategies to modify the neural networks underpinning tinnitus perception and distress.

## Data availability statement

The original contributions presented in the study are included in the article/Supplementary material, further inquiries can be directed to the corresponding author/s.

## Ethics statement

The studies involving human participants were reviewed and approved by University of Auckland Human Participants Ethics Committee. The patients/participants provided their written informed consent to participate in this study.

## Author contributions

GS: conceptualization, funding acquisition, and draft manuscript. GS and PS: methodology, analysis, and writing-review and editing. PS: data collection and project administration. Both authors contributed to the article and approved the submitted version.

## Funding

This study was supported by the Return on Science, Auckland UniServices limited.

## Conflict of interest

Author GS is a founder and scientific officer for TrueSilence a Spinout company of the University of Auckland and has a financial interest in TrueSilence. The remaining author declares that the research was conducted in the absence of any commercial or financial relationships that could be construed as a potential conflict of interest.

## Publisher's note

All claims expressed in this article are solely those of the authors and do not necessarily represent those of their affiliated organizations, or those of the publisher, the editors and the reviewers. Any product that may be evaluated in this article, or claim that may be made by its manufacturer, is not guaranteed or endorsed by the publisher.

## References

[1] McCormack A Edmondson-Jones M Somerset S Hall D. A systematic review of the reporting of tinnitus prevalence and severity. Hear Res. (2016) 337:70–9. 10.1016/j.heares.2016.05.009 27246985

[2] Bhatt JM Bhattacharyya N Lin HW. Relationships between tinnitus and the prevalence of anxiety and depression. Laryngoscope. (2017) 127:466–9. 10.1002/lary.26107 27301552 PMC5812676

[3] Theodoroff SM Lewis MS Folmer RL Henry JA Carlson KF. Hearing impairment and tinnitus: prevalence, risk factors, and outcomes in US service members and veterans deployed to the Iraq and Afghanistan wars. Epidemiol Rev. (2015) 37:71–85. 10.1093/epirev/mxu005 25600417

[4] Shahsavarani S Khan RA Husain FT. Tinnitus and the brain: a review of functional and anatomical magnetic resonance imaging studies. Perspect ASHA Spec Interest Groups. (2019) 4:896–909. 10.1044/2019_PERS-SIG6-2019-0001

[5] Searchfield G Kobayashi K Sanders M. An adaptation level theory of tinnitus audibility. Front Syst Neurosci. (2012) 6:46. 10.3389/fnsys.2012.00046 22707935 PMC3374480

[6] Durai M Kobayashi K Searchfield G. A preliminary examination of the roles of contextual stimuli and personality traits under the adaptation level theory model of tinnitus. Acta Acust United Acust. (2015) 101:543–51. 10.3813/AAA.918851

[7] Durai M Searchfield G. Anxiety and depression, personality traits relevant to tinnitus: a scoping review. Int J Audiol. (2016) 55:605–15. 10.1080/14992027.2016.1198966 27387463

[8] Searchfield GD. Tinnitus what and where: an ecological framework. Front Neurol. (2014) 5:271. 10.3389/fneur.2014.00271 25566177 PMC4266022

[9] De Ridder D Elgoyhen AB Romo R Langguth B. Phantom percepts: tinnitus and pain as persisting aversive memory networks. Proc Natl Acad Sci USA. (2011) 108:8075–80. 10.1073/pnas.1018466108 21502503 PMC3100980

[10] De Ridder D Vanneste S Congedo M. The distressed brain: a group blind source separation analysis on tinnitus. PLoS ONE. (2011) 6:e24273. 10.1371/journal.pone.0024273 21998628 PMC3188549

[11] Kleinjung T Langguth B. Avenue for future tinnitus treatments. Otolaryngol Clin N Am. (2020) 53:667–83. 10.1016/j.otc.2020.03.013 32381341

[12] Landry EC Sandoval XCR Simeone CN Tidball G Lea J Westerberg BD. Systematic review and network meta-analysis of cognitive and/or behavioral therapies (CBT) for tinnitus. Otol Neurotol. (2020) 41:153–66. 10.1097/MAO.0000000000002472 31743297

[13] Sereda M Xia J El Refaie A Hall DA Hoare DJ. Sound therapy (using amplification devices and/or sound generators) for tinnitus. Cochrane Database Syst Rev. (2018) 12:CD013094. 10.1002/14651858.CD013094.pub2 30589445 PMC6517157

[14] Hall DA Pierzycki RH Thomas H Greenberg D Sereda M Hoare DJ. Systematic evaluation of the T30 neurostimulator treatment for tinnitus: a double-blind randomised placebo-controlled trial with open-label extension. Brain Sciences. (2022) 12:317. 10.3390/brainsci12030317 35326273 PMC8946033

[15] Sereda M Smith S Newton K Stockdale D. Mobile apps for management of tinnitus: users' survey, quality assessment, and content analysis. JMIR mhealth uhealth. (2019) 7:e10353. 10.2196/10353 30672739 PMC6364200

[16] Scherer RW Formby C Tinnitus Retraining Therapy Trial Research Group. Effect of tinnitus retraining therapy vs standard of care on tinnitus-related quality of life: a randomized clinical trial. JAMA Otolaryngol Head Neck Surg. (2019) 145:597–608. 10.1001/jamaoto.2019.0821 31120533 PMC6547112

[17] Conlon B Langguth B Hamilton C Hughes S Meade E Connor CO. Bimodal neuromodulation combining sound and tongue stimulation reduces tinnitus symptoms in a large randomized clinical study. Sci Transl Med. (2020) 12:eabb2830. 10.1126/scitranslmed.abb2830 33028707

[18] Jastreboff PJ Hazell JWP. Treatment of tinnitus based on a neurophysiological model. In: Vernon JA editor. Tinnitus—Treatment and Relief. Boston, MA: Allyn and Bacon (1998).

[19] Searchfield GD Linford T Durai M. Sound therapy and aural rehabilitation for tinnitus: a person centred therapy framework based on an ecological model of tinnitus. Disabil Rehabil. (2019) 41:1966–73. 10.1080/09638288.2018.1451928 29571274

[20] Searchfield GD Durai M Linford T. A state-of-the-art review: personalization of tinnitus sound therapy. Front Psychol. (2017) 8:1599. 10.3389/fpsyg.2017.01599 28970812 PMC5609106

[21] Cederroth CR Gallus S Hall DA Kleinjung T Langguth B Maruotti A. Towards an understanding of tinnitus heterogeneity. Front Aging Neurosci. (2019) 11:53. 10.3389/fnagi.2019.00053 30941029 PMC6433929

[22] Searchfield GD Sanders PJ Doborjeh Z Doborjeh M Boldu R Sun K. A state-of-art review of digital technologies for the next generation of tinnitus therapeutics. Front Dig Health. (2021) 3:724370. 10.3389/fdgth.2021.724370 34713191 PMC8522011

[23] Searchfield G Kobayashi K Proudfoot K Tevoitdale H Irving S. The development and test–retest reliability of a method for matching perceived location of tinnitus. J Neurosci Methods. (2015) 256:1–8. 10.1016/j.jneumeth.2015.07.027 26306658

[24] Searchfield GD. A client oriented scale of improvement in tinnitus for therapy goal planning and assessing outcomes. J Am Acad Audiol. (2019) 30:327–37. 10.3766/jaaa.17119 30461417

[25] Searchfield GD Boone M Bensam J Durai M Hodgson S-A Linford T. A proof-of-concept study of the benefits of a single-session of tinnitus instruction and counselling with homework on tinnitus. Int J Audiol. (2020) 59:374–82. 10.1080/14992027.2020.1719436 32011194

[26] Barozzi S Del Bo L Crocetti A Dyrlund O Passoni S Zolin A. A comparison of nature and technical sounds for tinnitus therapy. Acta Acust United Acust. (2016) 102:540–6. 10.3813/AAA.918971

[27] Durai M Searchfield GD. A mixed-methods trial of broad band noise and nature sounds for tinnitus therapy: group and individual responses modeled under the adaptation level theory of tinnitus. Front Aging Neurosci. (2017) 9:44. 10.3389/fnagi.2017.00044 28337139 PMC5343046

[28] Durai M Kobayashi K Searchfield GD. A feasibility study of predictable and unpredictable surf-like sounds for tinnitus therapy using personal music players. Int J Audiol. (2018) 57:707–13. 10.1080/14992027.2018.1476783 29806782

[29] Searchfield GD Kobayashi K Hodgson S-A Hodgson C Tevoitdale H Irving S. Spatial masking: development and testing of a new tinnitus assistive technology. Assist Technol. (2016) 28:115–25. 10.1080/10400435.2015.1110214 26817495

[30] Durai M Sanders P Doborjeh Z Doborjeh M Wendt A Kasabov N. Prediction of tinnitus masking benefit within a case series using a spiking neural network model. Prog Brain Res. (2021) 260:129–65. 10.1016/bs.pbr.2020.08.003 33637215

[31] Searchfield GD Morrison-Low J Wise K. Object identification and attention training for treating tinnitus. Prog Brain Res. (2007) 166:441–60. 10.1016/S0079-6123(07)66043-9 17956809

[32] Wise K Kobayashi K Searchfield G. Feasibility study of a game integrating assessment and therapy of tinnitus. J Neurosci Methods. (2015) 249:1–7. 10.1016/j.jneumeth.2015.04.002 25863140

[33] Wise K Kobayashi K Magnusson J Welch D Searchfield GD. Randomized controlled trial of a perceptual training game for tinnitus therapy. Games Health J. (2016) 5:141–9. 10.1089/g4h.2015.0068 26910854

[34] Searchfield GD. Sense and sensibility: a review of the behavioral neuroscience of tinnitus sound therapy and a new typology. Behav Neurosci Tinnitus. (2020) 51:213–47. 10.1007/7854_2020_183 33547596

[35] Meikle MB Henry JA Griest SE Stewart BJ Abrams HB McArdle R. The tinnitus functional index: development of a new clinical measure for chronic, intrusive tinnitus. Ear Hear. (2012) 33:153–76. 10.1097/AUD.0b013e31822f67c0 22156949

[36] Searchfield G Warr A Kuklinski J Purdy S editors. Digital instruments for tinnitus: mixing point identification and threshold-adjusted noise. In: Proceedings of the Seventh International Tinnitus Seminar. Perth, WA: The University of Western Australia (2002).

[37] Langguth B Goodey R Azevedo A Bjorne A Cacace A Crocetti A. Consensus for tinnitus patient assessment and treatment outcome measurement: tinnitus research initiative meeting, regensburg, July 2006. Prog Brain Res. (2007) 166:525–36. 10.1016/S0079-6123(07)66050-6 17956816 PMC4283806

[38] Chandra N Chang K Lee A Shekhawat GS Searchfield GD. Psychometric validity, reliability, and responsiveness of the tinnitus functional index. J Am Acad Audiol. (2018) 29:609–25. 10.3766/jaaa.16171 29988009

[39] Carhart R Jerger JF. Preferred method for clinical determination of pure-tone thresholds. J Speech Hear Disord. (1959) 24:330–45. 10.1044/jshd.2404.330

[40] Poirier-Quinot D Katz BFG. The anaglyph binaural audio engine. In: Audio Engineering Society Convention 144. Milan: Audio Engineering Society (2018).

[41] Brooke J. Sus: a quick and dirty usability. In: Jordan PW Thomas B Weerdmeester BA McClelland IL editors. Usability Evaluation in Industry. London: CRC Press (1996). p. 189–94.

[42] Zhou L Bao J Setiawan IMA Saptono A Parmanto B. The mHealth app usability questionnaire (MAUQ): development and validation study. JMIR mHealth uHealth. (2019) 7:e11500. 10.2196/11500 30973342 PMC6482399

[43] Aydin N Searchfield GD. Changes in tinnitus and physiological biomarkers of stress in response to short-term broadband noise and sounds of nature. Complement Ther Med. (2019) 46:62–8. 10.1016/j.ctim.2019.07.018 31519289

[44] Hall DA Smith H Hibbert A Colley V Haider HF Horobin A. The COMiT'ID study: developing core outcome domains sets for clinical trials of sound-, psychology-, and pharmacology-based interventions for chronic subjective tinnitus in adults. Trends Hear. (2018) 22:2331216518814384. 10.1177/2331216518814384 30488765 PMC6277759

[45] Barros Suzuki FAD Suzuki FA Yonamine FK Onishi ET Penido NO. Effectiveness of sound therapy in patients with tinnitus resistant to previous treatments: importance of adjustments. Braz J Otorhinolaryngol. (2016) 82:297–303. 10.1016/j.bjorl.2015.05.009 26541232 PMC9444648

