# Supplementary material for: A randomized single-blind controlled trial of a prototype digital polytherapeutic for tinnitus
Source: Front Neurol. 2022 Aug 5;13:958730. doi: 10.3389/fneur.2022.958730 (PMC9389120; doi:10.3389/fneur.2022.958730)
Supplement: Supplementary file 1 [file Data_Sheet_1.pdf]

Supplement Table 1. Additional participant characteristics

		PP		ITT	
**Measure**		**USL**	**WN**	**USL**	**WN**
**Number of participants**		31	30	50	48
**Hand**	Right	20	21	33	36
	Left	6	5	9	8
	Ambidextrous	5	4	8	4
**Family history**	No	23	20	33	38
	Yes	7	11	17	10
**Onset**	Gradual	15	18	27	31
	Sudden	16	12	23	17
**Cause**	Loud blast	8	7	9	9
	Hearing loss	5	6	10	8
	Stress	7	2	8	6
	Head Trauma	2	4	3	0
	Other	9	15	20	21
**Pulses**	No	29	22	42	34
	Yes with heart beat	2	3	4	7
	Yes not with heart beat	0	5	4	7
**Manifest**	Intermittent	3	4	5	5
	Constant	28	26	45	43
**Loudness varies**	No	9	11	17	19
	Yes	22	19	33	29
**Tinnitus Sound**	Tone	15	12	14	17
	Noise	7	12	11	9
	Cricket	9	4	24	19
**Pitch**	Very high	9	9	13	12
	High	21	17	33	31
	Medium	1	3	4	4
**Treatment**	None	19	22	33	37
	One	7	3	10	3
	Several	5	3	7	6
	many	0	2	0	2
**Enirvonmental sounds mask**	No	3	2	6	2
	Yes	20	19	32	30
	Don't know	8	9	12	16
**Loud sounds worsen**	No	11	10	20	18
	Yes	14	7	18	14
	Don't know	6	13	12	16
**Head/neck movement changes**	No	23	23	34	38
	Yes	8	7	17	10

**Nap effect**	Worst	5	1	5	2
	Better	2	2	2	4
	No effect	24	27	43	42
**Sleep relationship**	No	9	11	13	15
	Yes	4	3	5	8
	Don't know	18	16	32	25
**Stress**	Worse	22	17	34	28
	No effect	9	13	16	20
**Medication effect**	No	30	26	49	40
	Yes	1	4	1	7
**Hyperacusis**	Never	0	1	6	4
	Rare	5	5	13	8
	Sometimes	10	16	16	24
	Usually	5	5	7	9
	Always	6	3	8	3
**Headache**	No	20	19	18	18
	Yes	11	11	32	29
**Vertigo**	No	20	20	34	31
	Yes	11	10	16	16
**TMJ**	No	28	28	46	44
	Yes	3	2	4	3
**Neck pain**	No	22	19	34	30
	Yes	9	11	16	17
**Other pain**	No	26	27	40	40
	Yes	5	3	10	8
**Current psychological care**	No	30	28	49	44
	Yes	1	2	1	4

Supplement Table 2. Summary ANOVA statistics for Tinnitus Functional index. Statistically significant differences are shaded grey.

TFI 2-Way ANOVA, mean (standard deviation)							
		Session				Statistic	
Measure	Group	Screening	Baseline	6 weeks	12 weeks	Session	Interaction
Total	USL	59.77 (10.91)	57.39 (11.82)	41.03 (19.09)	39.56 (22.09)	F (1.603, 94.57) = 34.88, P<0.0001	F (3, 177) = 1.516, n.s
	WN	61.19 (13.40)	57.87 (12.79)	47.09 (20.70)	47.75 (21.11)		
Intrusive	USL	65.05 (15.05)	65.05 (16.62)	52.47 (17.28)	46.69 (24.19)	F (1.918, 113.2) = 24.29, P<0.0001	F (3, 177) = 0.732, n.s
	WN	70.56 (15.19)	68.67 (12.67)	56.67 (19.69)	56.67 (20.55)		
Control	USL	72.04 (15.48)	66.45 (14.60)	52.69 (24.85)	45.48 (26.96)	F (1.874, 110.6) = 28.09, P<0.0001	F (3, 177) = 0.890, n.s
	WN	71.33 (14.05)	67.33 (19.39)	56.44 (25.19)	53.22 (23.98)		
Cognitive	USL	50.00 (18.20)	48.06 (21.13)	35.16 (20.92)	31.18 (25.22)	F (2.265, 133.6) = 18.54, P<0.0001	F (3, 177) = 0.539, n.s
	WN	52.44 (19.92)	52.00 (17.93)	39.56 (25.59)	40.11 (23.51)		
Sleep	USL	51.61 (25.34)	53.87 (26.56)	34.09 (29.65)	35.05 (29.91)	F (2.212, 130.5) = 17.57, P<0.0001	F (3, 177) = 2.117, n.s
	WN	58.33 (28.68)	53.22 (28.40)	46.33 (30.73)	45.22 (30.90)		
Auditory	USL	61.4 (22.06)	60.65 (19.86)	41.51 (23.80)	40.11 (24.00)	F (2.258, 133.2) = 22.24, P<0.0001	F (3, 177) = 3.020, P=0.0312
	WN	59.00 (28.98)	59.33 (28.18)	49.22 (27.75)	50.22 (31.26)		
Relax	USL	73.01 (19.90)	69.57 (20.78)	50.86 (25.91)	50.32 (26.80)	F (1.744, 102.9) = 22.89, P<0.0001	F (3, 177) = 1.608, n.s
	WN	72.67 (19.05)	68.00 (18.06)	57.89 (25.26)	58.78 (24.75)		
QOL	USL	51.45 (21.05)	46.85 (20.62)	31.77 (21.45)	31.61 (25.10)	F (2.221, 131.1) = 21.92, P<0.0001	F (3, 177) = 0.791, n.s
	WN	52.17 (20.40)	53.17 (21.95)	39.58 (25.43)	39.42 (25.69)		
Emotion	USL	57.63 (23.10)	50.97 (23.23)	35.81 (25.09)	35.16 (29.95)	F (2.019, 119.1) = 25.89, P<0.0001	F (3, 177) = 0.580, n.s
	WN	56.00 (22.43)	53.00 (23.07)	37.56 (27.12)	40.89 (25.95)		

Supplement Table 3. Summary post-hoc Dunnett's multiple comparison test statistics within group compared to baseline for TFI. Statistically significant differences are shaded grey.

		USL		WN	
Measure	Session	Difference to BL	Statistic	Difference to BL	Statistic
Total	Screening	-2.37	q(30)=1.920, n.s	-3.32	q(29)=2.888, n.s
	6 weeks	16.36	q(30)=5.071, P<0.0001	10.77	q(29)=3.185, p=0.0095
	12 weeks	17.83	q(30)=4.998, P<0.0001	10.12	q(29)=2.595, p=0.0386
Intrusive	Screening	0.00	q(30)=0.000, n.s	-1.89	q(29)=0.7067, n.s
	6 weeks	12.58	q(30)=4.559, P=0.0002	12.00	q(29)=3.557, p=0.0037
	12 weeks	18.36	q(30)=4.247, P<0.0006	12.00	q(29)=2.948, p=0.0386
Control	Screening	-5.59	q(30)=3.256, P=0.0077	-4.00	q(29)=1.642, n.s
	6 weeks	13.76	q(30)=3.004, P=0.0145	10.89	q(29)=2.378, n.s
	12 weeks	20.97	q(30)=4.663, P=0.0002	14.11	q(29)=2.888, p=0.0195
Cognitive	Screening	-1.94	q(30)=0.668, n.s	-0.44	q(29)=0.156, n.s
	6 weeks	12.90	q(30)=3.153, P=0.0100	12.44	q(29)=3.214, p=0.0088
	12 weeks	16.88	q(30)=3.946, P=0.0013	11.89	q(29)=2.619, p=0.0365
Sleep	Screening	2.26	q(30)=0.912, n.s	-5.11	q(29)=1.826, n.s
	6 weeks	19.78	q(30)=4.790, P=0.0001	6.89	q(29)=1.815, n.s
	12 weeks	18.82	q(30)=3.745, P=0.0022	8.00	q(29)=2.317, n.s
Auditory	Screening	-0.75	q(30)=0.2723, n.s	0.33	q(29)=0.1557, n.s
	6 weeks	19.14	q(30)=4.405, P=0.0004	10.11	q(29)=3.089, p=0.0120
	12 weeks	20.54	q(30)=2.314, P=0.0001	9.11	q(29)=2.314, p=0.0713
Relax	Screening	-3.44	q(30)=1.479, n.s	-4.67	q(29)=1.443, n.s
	6 weeks	18.71	q(30)=4.177, P=0.0007	10.11	q(29)=2.253, n.s
	12 weeks	19.25	q(30)=4.365, P=0.0004	9.22	q(29)=1.774, n.s
QOL	Screening	-4.60	q(30)=1.430, n.s	1.00	q(29)=0.3291, n.s

	6 weeks	15.08	q(30)=3.456, P=0.0004	13.58	q(29)=3.322, P=0.0067
	12 weeks	15.24	q(30)=4.104, P=0.0023	13.75	q(29)=2.787, P=0.0248
Emotional	Screening	-6.67	q(30)=2.322, n.s	-3.00	q(29)=0.927, n.s
	6 weeks	15.16	q(30)=3.874, P=0.0015	15.44	q(29)=3.306, p=0.0070
	12 weeks	15.81	q(30)=3.629, P=0.0029	12.11	q(29)=2.211, p=0.0883

Supplement Table 4. Summary Friedman test statistics for rating scales. Statistically significant differences are shaded grey.

Rating scales Friedman test within intervention, median						
Measure	Intervention	Screening	Baseline	6 weeks	12 weeks	Session
Problem	USL	3	3	3	3	$\chi2$=19.66, P=0.0002
	WN	4	3	3	3	$\chi2$=8.141, P=0.0432
Strong	USL	8	7	6	5	$\chi2$=17.02, P=0.0007
	WN	8	8	7	7.5	$\chi2$=8.056, P=0.0449
Uncomfortable	USL	8	7	7	6	$\chi2$=25.98, P<0.0001
	WN	8	8	7.5	7	$\chi2$=11.75, P=0.0083
Annoyance	USL	8	7	5	6	$\chi2$=25.05, P<0.0001
	WN	8	8	6.5	7	$\chi2$=11.38, P=0.0099
Ignore	USL	8	8	5	5	$\chi2$=25.78, P<0.0001
	WN	7.5	8	6.5	6	$\chi2$=9.037, P=0.0288
Unpleasant	USL	8	8	6	5	$\chi2$=30.81, P<0.0001
	WN	8	8	7	7	$\chi2$=20.43 P=0.0001

Supplement Table 5. Summary post-hoc Dunn's multiple comparison test statistics within group compared to baseline for rating scales. Statistically significant differences are shaded grey.

		USL		WN	
Rating	Session	RSum difference to BL	Statistic	Rsum difference to BL	Statistic
Problem	Screening	-19.00	Z=1.869, n.s	-4.00	Z=0.400, n.s
	6 weeks	13.00	Z=1.279, n.s	16.00	Z=1.600, n.s
	12 weeks	12.00	Z=1.180, n.s	10.00	Z=1.000, n.s
Strong	Screening	-6.00	Z=1.869, n.s	-5.00	Z=0.5000, n.s
	6 weeks	22.50	Z=2.213, n.s	15.00	Z=1.5000, n.s
	12 weeks	25.50	Z=2.509, P=0.1854	16.00	Z=1.6000, n.s
Uncomfortable	Screening	-20.00	Z=1.967, n.s	-11.00	Z=1.100, n.s
	6 weeks	23.50	Z=2.312, n.s	9.00	Z=0.900, n.s
	12 weeks	18.50	Z=1.820, n.s	18.00	Z=1.800, n.s
Annoyance	Screening	-7.50	Z=0.7378, n.s	-6.00	Z=0.600, n.s
	6 weeks	27.50	Z=2.705, P=0.0205	21.00	Z=2.100, n.s
	12 weeks	32.00	Z=3.148, P=0.0049	17.00	Z=1.700, n.s
Ignore	Screening	-10.00	Z=0.9837, n.s	-7.50	Z=0.7500, n.s
	6 weeks	26.00	Z=2.558, P=0.316	14.50	Z=1.450, n.s
	12 weeks	30.00	Z=2.951, P=0.0095	15.00	Z=01.500, n.s
Unpleasant	Screening	-18.00	Z=1.771, n.s	-2.00	Z=0.2000, n.s
	6 weeks	27.50	Z=2.705, P=0.0205	23.50	Z=2.350, n.s
	12 weeks	24.50	Z=2.410, P=0.0478	30.50	Z=3.050, P=0.0069

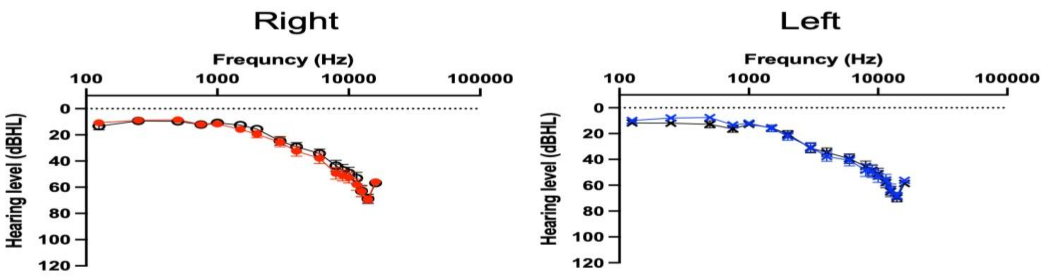

SFIG 1

Supplement Figure 1. Audiogram for intent-to-treat participants. Color USL group (n=50) black WN group (n=48). Mean thresholds and standard error bars are shown.
